# Intraoperative Radiation for Breast Cancer with Intrabeam™: Factors Associated with Decreased Operative Times in Patients Having IORT for Breast Cancer

**DOI:** 10.3389/fonc.2017.00237

**Published:** 2017-10-09

**Authors:** Stephanie A. Valente, Alicia Fanning, Robyn A. Stewart, Sharon Grundfest, Rahul D. Tendulkar, Sheen Cherian, Chirag Shah, Chao Tu, Courtney Yanda, Diane Radford, Zahraa Al-Hilli, Stephen R. Grobmyer

**Affiliations:** ^1^Division of Breast Services, Department of General Surgery, Cleveland Clinic, Cleveland, OH, United States; ^2^Department of Radiation Oncology, Cleveland Clinic, Cleveland, OH, United States; ^3^Quantitative Health Sciences, Cleveland Clinic, Cleveland, OH, United States

**Keywords:** intraoperative radiation therapy, surgery, operation, time, applicator, experience, radiation oncology, breast surgery

## Abstract

**Introduction:**

Intraoperative radiation with Intrabeam™ (IORT) for breast cancer is a newer technology recently implemented into the operating room (OR). This procedure requires time and coordination between the surgeon and radiation oncologist, who both perform their treatments in a single operative setting. We evaluated the surgeons at our center, who perform IORT and their OR times to examine changes in OR times following implementation of this new surgical procedure. We hypothesized that IORT is a technique for which timing could be improved with the increasing number of cases performed.

**Methods:**

A prospectively maintained IRB approved database was queried for OR times (incision and close) in patients who underwent breast conserving surgery (BCS), sentinel lymph node biopsy with and without IORT using the Intrabeam™ system at our institution from 2011 to 2015. The total OR times were compared for each surgeon individually and over time. Next, the OR times of each surgeon were compared to each other. Continuous variables were summarized and then a prediction model was created using IORT time, OR time, surgeon, and number of cases performed.

**Results:**

There were five surgeons performing IORT at our institution during this time period with a total of 96 cases performed. There was a significant difference observed in baseline surgeon-specific OR time for BSC (*p* = 0.03) as well as for BCS with IORT (*p* < 0.05), attributable to surgeon experience. The average BCS times were faster than the BCS plus IORT procedure times for all surgeons. The overall mean OR time for the entire combined surgical and radiation procedure was 135.5 min. The most common applicator sizes used were the 3.5 and 4 cm, yielding an average 21 min IORT time. Applicator choice did not differ over time (*p* = 0.189). After adjusting for IORT time and surgeon, the prediction model estimated that surgeons decreased the total BCS plus IORT OR time at a rate of −4.5 min per each additional 10 cases performed.

**Conclusion:**

Surgeon experience and applicator size are related to OR times for performing IORT for breast cancer. OR time for IORT in breast cancer treatment can be improved over time, even among experienced surgeons.

## Introduction

Adjuvant intraoperative radiation with the Intrabeam™ system (IORT) is an excellent therapeutic option for selected patients with breast cancer. Evidence to support the use of IORT comes from the TARGIT-A randomized trial showing that for early stage breast cancer, risk of local recurrence with IORT performed at the time of lumpectomy surgery is not statistically different than whole breast radiation (WBRT) (2.1% with IORT compared to 1.1% with WBRT, *p* = 0.31) (1, 2). This was confirmed with the large North American TARGIT-Retrospective Study supporting similar low local recurrence rates and showing utilization of IORT for breast cancer treatment is growing in North America and globally (3).

In the treatment of breast cancer, IORT has been shown to have many advantages for patients including convenience, improved quality of life (4), and lower cost compared to more traditional treatments (5). However, the use of IORT as adjunct for breast cancer treatment does prolong operating room (OR) times for patients having breast-conserving surgery. OR time is a costly and precious resource within health-care systems (6). There is increasing pressure on value-based health-care delivery and efficient care (7). Little is known about the impact of performing IORT with Intrabeam™ on OR times and factors associated with decreased operative times (8).

We hypothesized that the time to perform IORT in combination with breast conserving surgery (BCS) can be decreased with the increasing number of cases performed. In this study, we sought to document OR times associated with performance of BCS and IORT and analyze factors associated with operative time. This information is critical for assigning appropriate resources, OR allocations, and for optimizing efficient use of ORs within centers offering IORT for the treatment of breast cancer.

## Materials and Methods

A prospectively maintained database was queried for OR times (incision and close) in patients who underwent lumpectomy, sentinel lymph node biopsy with and without IORT using the Intrabeam™ system (Carl Zeiss AG: Oberkochen, Germany) at our institution from 2011 to 2015. Only IORT cases performed as a unilateral procedure at the time of initial lumpectomy were included. OR time was defined from incision to closure in this study. OR times included combined performance of lumpectomy, sentinel node biopsy, and IORT. In our practice, frozen section analysis was performed on axillary sentinel nodes in patients having IORT. The size applicator used for IORT can vary at our institution from 2.5 to 5 cm. Surgeons choose an applicator size based on the size of the lumpectomy cavity, which is a result of the size tumor removed. IORT was performed by 1 of 2 radiation oncologists who were present in the OR for these procedures. IORT delivery time was calculated based on the size applicator used.

Surgeons who performed conserving surgery (BCS) operation with IORT at our institution during this time period were identified. The IORT applicator sizes used and total OR times for all the identified surgeons were then analyzed. As a baseline control for OR time, surgeons individual OR times for BCS alone during the study period were averaged and compared. Then, their individual OR times for BCS with IORT were analyzed and compared. Next, the OR times for each surgeon who had performed greater than 10 IORT cases during the study period were evaluated. Statistical analysis was performed using the Kruskal–Wallis test and repeated measures ANOVA. Continuous variables were summarized and then a prediction model was created using IORT time, OR time, surgeon, and number of cases performed. Since IORT time is a standard prescribed time based on the size applicator used, this was controlled for in the prediction model. A *p*-value < 0.05 was considered statistically significant. This study was carried out in accordance with the recommendations of the Cleveland Clinic Foundation Institutional Review Board, under which it was reviewed and approved. A waiver of informed consent was granted, as all patient data were de-identified.

## Results

There were five surgeons identified performing IORT at our institution during this time period with a total of 96 cases performed. The most common applicator sizes used were the 3.5 cm (33%) and the 4 cm (36%), yielding an average 21-min IORT time (Table 1). Applicator size did not differ over time (*p* = 0.189). Longer OR times were significantly associated with use of larger applicator sizes, as a longer time is required to deliver the prescribed dose of radiation (*p* < 0.0001).

**Table 1:** Applicator sizes used for IORT cases with Intrabeam™ in this series.

Applicator size	Percentage (%) of cases	Number of cases performed *N* = 96
2.5	3	3
3	9	9
3.5	33	32
4	35	34
4.5	13	12
5	6	6

The overall mean OR time for all surgeons for the entire combined BCS and IORT procedure was 135.5 min (102–173 min). There was a significant difference observed in surgeon specific mean OR time for BCS with IORT (131 vs 185 min, *p* = 0.03) (Table 2). Only one of the five surgeons had significant prior experience with performing IORT (Surgeon E). This surgeon with the greatest IORT experience had the lowest mean operating time (131 min), which was significantly lower than the surgeon with least IORT experience (185 min, *p* = 0.02).

**Table 2:** Operating room times for five different surgeons performing BCS IORT with Intrabeam™ in this series.

Surgeon	No. procedures in series	Minimum time (min)	Mean time (min)	Maximum time (min)
A	2	170	185	200
B	5	117	184	218
C	14	65	139	228
D	26	127	176	261
E^a^	49	83	131	210

*^a^The surgeon with prior significant experience performing IORT*.

**Figure 1:**
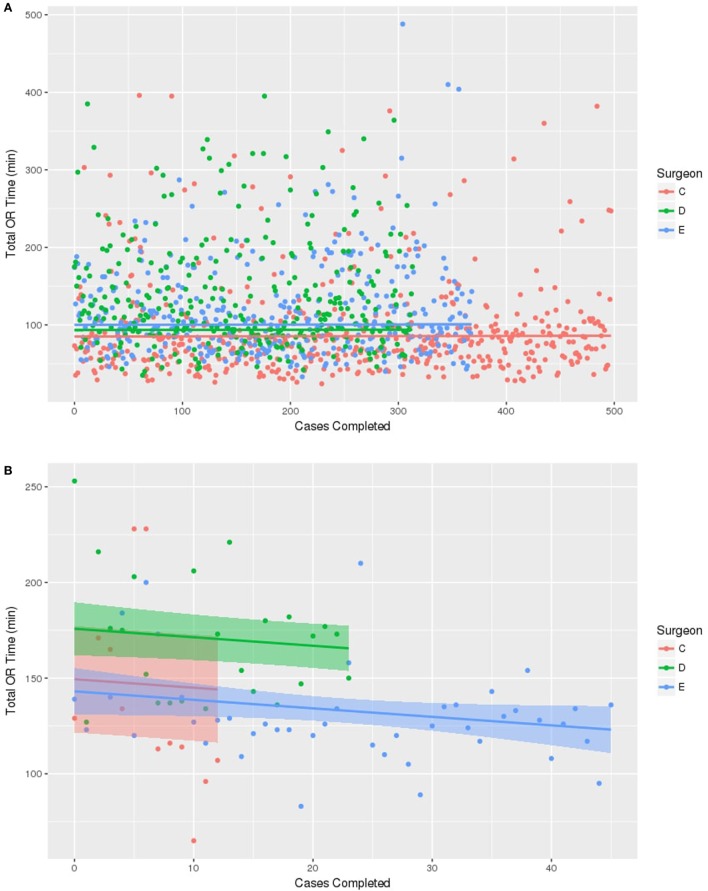
Prediction line graphs for surgeon operating room (OR) time for cases completed. The model includes the three surgeons who had performed greater than 10 cases. **(A)** No change in time observed among any of the three surgeons in OR time for breast conserving surgery without IORT. **(B)** Reduction in OR time is associated with increasing surgeon number of cases with IORT using Intrabeam™ system. Every surgeon decreased OR time by −4.5 min for each 10 cases performed.

For the purpose of prediction modeling, only surgeons with at least 10 operations performed were used, giving a total of three surgeons evaluated in this analysis (Figure 1). First, a control group was created using the BCS only OR times for the three surgeons during 2011–2015. Figure 1A shows that at baseline, the three surgeons did differ significantly in their average overall BCS only OR times [(Surgeon C 86.8 min, D 95.2 min, E 101.8 min), *p* < 0.001] and this difference was maintained over the study period without significant improvement in OR time over time. Next, BCS with IORT times for the three surgeons were analyzed (Figure 1B). This demonstrates that there were baseline differences among surgeons in total OR time for BCT with IORT (131 vs 176 min, *p* < 0.001). Surgeon E, with the prior IORT experience, had the fastest average BCS with IORT time (131 min). Similar to the BCS only procedure, the surgeon’s individual OR time differences for BCS with IORT were maintained over time. Interestingly, for BCS with IORT, despite differences in individual OR times, all surgeons (C, D, and E) regardless of prior IORT OR experience were found to have significantly decreasing operating time with an increasing number of cases performed over the study period (*p* = 0.02). After adjusting for IORT time and surgeon, the prediction model estimated that each surgeon decreased the total OR time for BCS with IORT at a rate of −4.5 min per each additional 10 cases performed.

## Discussion

This study documents that total OR time with IORT Intrabeam™ does decrease with increasing experience with the technique. The decreased time is likely related to surgeons and operating teams becoming more familiar with the procedure and improved coordination of the required steps to complete the breast conserving surgery, sentinel node biopsy, and IORT. It is noteworthy that even a highly experienced IORT surgeon (Table 2, Surgeon E) can improve OR times as experience with an operating team and radiation oncology team grows. The reported times in this series compare favorably to operating times reported utilizing an alternative form of IORT, which was reported to be 140 min (9). We hypothesize that while the delivery of radiation is longer with Intrabeam™ compared to other systems, the reproducible simplicity of setup and cavity preparation of Intrabeam™ accounts for the lower overall observed OR times with Intrabeam™.

We did not investigate the specific time of each step as it relates to the observed decrease in operative times. However, there are several specific areas in the procedure where efficiencies can be realized to decrease total operation time such as: surgeon technique including performance of intraoperative radiation and purse-string suture placement; equipment setup and having the proper equipment in the OR by OR staff (shielding drapes, ultrasound, etc.); and coordination of the arrival of the radiation oncologist and physicist to coincide with the start of the IORT portion of the case.

In this series, all patients had frozen section performed as part of the procedure as our programmatic approach during the time of the study was to not perform IORT in patients with axillary metastasis. There is a potential to reduce OR times further if this step was eliminated and patients were treated with IORT regardless of nodal status. IORT has been shown to be an effective boost replacement for patients requiring WBRT therapy (10). Other opportunities to improve OR times include: having standard operating teams and nursing teams who are familiar with the procedure, use of anticipatory paging of radiation oncology team members to avoid delays waiting for their arrival, and performing procedures at standard time and in a standard location.

The data in this report are important, as this is the first documentation of time associated with the performance of BCS and IORT using the Intrabeam™ system. These data can be used for planning operating time and resource allocation and serve as a benchmark for planning operating days for new teams adopting the Intrabeam™ system. Importantly, this series shows us that surgeons and treatment teams can become more efficient over time with IORT delivery as experience with the technique grows.

## Author Contributions

Each author has collaborated together to make contributions to conception and design, with the acquisition of data, and the analysis and interpretation of data. Each author has offered revisions and input on the manuscript. Each author gave their final approval of the submitted manuscript.

## Conflict of Interest Statement

ZEISS Meditech provides travel and education funding for SG. All other authors declare that the research was conducted in the absence of any commercial or financial relationships that could be construed as a potential conflict of interest.
